# Severe acute malnutrition and its associated factors among children under-five years: a facility-based cross-sectional study

**DOI:** 10.1186/s12887-020-02154-1

**Published:** 2020-05-26

**Authors:** Umesh Ghimire, Binod Kumar Aryal, Ankush Kumar Gupta, Suman Sapkota

**Affiliations:** 1 New ERA, Kalopul, Rudramati Marga, Kathmandu, 44600 Nepal; 2 Partnership for Social Development, Kathmandu, 446006 Nepal

**Keywords:** Severe acute malnutrition, Under-five children, Nutritional status, Mid-upper arm circumference, Nepal

## Abstract

**Background:**

Despite consistent efforts to enhance child nutrition, poor nutritional status of children continues to be a major public health problem in Nepal. This study identified the predictors of severe acute malnutrition (SAM) among children aged 6 to 59 months in the two districts of Nepal.

**Methods:**

We used data from a cross-sectional study conducted among 6 to 59 months children admitted to the Outpatient Therapeutic Care Centers (OTCC). The nutritional status of children was assessed using mid-upper arm circumference (MUAC) measurement. To determine which variables predict the occurrence of SAM, adjusted odds ratio was computed using multivariate logistic regression and *p*-value < 0.05 was considered as significant.

**Results:**

Out of 398 children, 5.8% were severely malnourished and the higher percentage of female children were malnourished. Multivariate analysis showed that severe acute malnutrition was significantly associated with family size (five or more members) (Adjusted Odds Ratio [AOR]: 3.96; 95% Confidence Interval [CI]: 1.23–12.71). Children from severely food insecure households (AOR: 4.04; 95% CI: 1.88–10.53) were four times more likely to be severely malnourished. Higher odds of SAM were found among younger age-group (AOR: 12.10; 95% CI: 2.06–71.09) children (0–12 vs. 24–59 months).

**Conclusions:**

The findings of this study indicated that household size, household food access, and the child’s age were the major predictors of severe acute malnutrition. Engaging poor families in kitchen gardening to ensure household food access and nutritious diet to the children, along with health education and promotion to the mothers of young children are therefore recommended to reduce child undernutrition.

## Background

Children’s right to have access to safe diet and adequate nutrition is undeniable, and fulfilment of their right is essential to attain the highest standard of health [1]. Children’s age under 59 months is the critical period for rapid physical growth as well as overall child development. Children suffer from various forms of undernutrition if the nutritional requirement is compromised. Undernutrition among children is a significant contributor to the global disease burden and a leading cause of child mortality worldwide [2]. Severe acute malnutrition (SAM) refers to the condition that is identified by the Mid-Upper Arm Circumference (MUAC) measurement of less than 115 mm or weight for height (wasting) less than minus 3SD z-score below the median in 6 to 59 months children [3]. Classification of mild, moderate, or severe undernutrition is based on anthropometric, biochemistry measurement, and clinical assessment [4]. Though the risk of death among children in the SAM category (MUAC < 115 mm) is closely associated with the severity of undernutrition, SAM children usually have an infirmed immune response that puts them at increased risk of dying [3, 5].

Globally, 7.5 million under-five children are wasted and 16.4 million are severely undernourished [6]. SAM contributes to over one million under-five deaths per annum. Survivors of acute malnourished children are at increased risk of developing stunting and various diseases, disorders, poor educational performance, and low productive life [7]. Child undernutrition is a critical public health issue ubiquitous in many developing countries where various infectious diseases are rampant [5, 8]. Several studies have showed the association between SAM and poverty [9, 10], large family size [11], low dietary diversity and unimproved sanitation, and hygiene [9, 12], exposure to pathogens, and recurrent infections [13, 14]. The prevalence of stunting wasting and underweight according to the 2016 Nepal Demographic and Health Survey (NDHS) was 36%, 10%, and 27%, respectively. The rate of stunting and underweight among under-five years children showed a gradual downward trend since 1996, however, the decline in wasting was minimal. The rate of wasting among under-five years children was higher in rural areas and in provinces 1 and 2 [15, 16]. Overall health indicators in province 2 are relatively low, especially among poorer households. The problem of undernutrition in province 2 is still high and poses challenges in the attainment of goals to improve the nutrition status of children [17].

Children are the most vulnerable population and are more likely to be undernourished during the emergency [18]. Several studies have discussed the possibility of treating SAM children at the community level in an emergency setting through outpatient therapeutic care centers (OTCC) [19, 20]. As per the protocol of Nepal Integrated Management of Acute Malnutrition (IMAM), a child with SAM can be treated in OTCC if no further medical complications are present [21, 22]. The treatment of SAM through community-level health facilities was possible because of the development of ready-to-use therapeutic foods (RUTF) and acceptance of MUAC for the screening of SAM [23]. MUAC is a widely used rapid nutritional assessment approach based on the assumption that it closely related to muscle mass to identify SAM in children [24].

Besides disruption of food supply chain and socio-demographic stability, natural calamities such as earthquake and floor, affect the systematic health services affecting health care utilization of mothers and children. During the monsoon season, districts in Eastern Terai (low land in province 1 and 2) are hard hit by flooding, disturbing infrastructure, and social phenomena. Heavy rainfall in August 2017 triggered flash flood and landslide in 32 Terai districts in Nepal including Bara and Jhapa. Food supply, and basic medical services were heavily interrupted due to widespread flooding [25]. In addition, nutritious food consumption by the children and mothers during this period was well below minimum standards due to the low availability of the ration and other food and non-food items [26].

There is a lack of evidence on the prevalence and determinants of SAM among children aged 6–59 months. To our best knowledge, this study is the first of its kind in Nepal to investigate the risk factors of SAM soon after an emergency. Therefore, the objective of the study was to identify the factors related to severe acute malnutrition and to generate comprehensive findings that could be helpful in planning nutrition programs in emergency settings in Nepal.

## Methods

### Study area

This study was conducted among children in two randomly selected Terai (low land) districts from province 1 (Jhapa) and province 2 (Bara). Jhapa district is better off than Bara district in terms of the human development index [27].

### Study settings and participants

A cross-sectional study was conducted among children 6–59 months from April to June 2018. The sample size for the study was calculated using the formula, *n* = Z^2^p(1 – p)/d^2^ where n is the number of samples required, Z is the value associated with the 95% confidence interval, d is the precision margin of error at 5%, and p is the prevalence of diseases. For this study, we assumed 14.4% prevalence rate of acute malnutrition, 5% margin of error, and 95% confidence level [15, 22]. The sample size of 197 plus an addition 10% non-response rate yielded a total sample size of 217 for each district. The final sample size after data cleaning and excluding incomplete data from the two districts was 398. OTCC in a district was considered as strata. All OTCCs, 16 from Jhapa and 19 from Bara, were included in the sampling frame and selected using a systematic sampling method. The required number of children was then divided by the number of OTCCs and selected consecutively after a fixed interval.

### Inclusion criteria

Eligible mothers of 6 to 59 months children attending OTCCs were selected and interviewed.

### Exclusion criteria

Children with known chronic illnesses, and congenital abnormality which affects the feeding pattern of the children were excluded from the study.

### Questionnaire

The structured questionnaire was developed based on the study objectives. Indicators related to household socio-economic characteristics, education level, toilet facility, and food security [28], nutritional assessment, breastfeeding status, were adopted from the 2016 NDHS questionnaire [15]. All the questions were pretested during the pilot test in a non-sampled OTCC.

### Outcome variable

Outcome of interest for this study was severe acute malnutrition among children aged between 6 to 59 months [3]. To identify acute malnutrition in children, measurement of MUAC less than 115 mm was used as cut-off criteria based on the 2007 WHO child growth standards [29].

### Independent variables

Independent variables were categorized into three levels; household factors, child factors, and maternal factors. A robust literature review was done to regroup the potential factors associated with SAM. Household factors included variables such as place of residence (urban and rural municipality), family type, family size, ethnicity, household income, availability of toilet facility at the household, possession of land size, kitchen garden, and household food insecurity. The family type was classified as nuclear and joint, and the family size was categorized into two categories i) 1 to 5 members, and ii) 5 or more members. The ethnicity variable was grouped into three categories: relatively advantaged (Brahmin/Chhetri), relatively disadvantaged (Janajati/Muslims), and Madhesi and other unidentified. Madhesi is a predominant caste in Nepal who reside in the Terai region [30]. The annual income of a household was asked with the respondent to determine the economic status of household. The household income was categorized based on annual family income standards as recommended by the National Bank (Nepal Rastriya Bank) [31]. The possession of land size was grouped into two categories: households having less than 0.5 hectors of land and households having 0.5 or more hectors of land. Household Food Insecurity Access Scale (HFIAS) measurement tool was used to collect information on food insecurity at household level developed by the Food and Nutrition Technical Assistance Project (FANTA) [28]. Child factors comprised of gender and age of child, number of children in the household variables. Birth order was categorized as first, second, and third, or more. Likewise, the birth interval of between child was classified into two categories; i) less than 2 years and ii) more than 2 years. Maternal level factors included mother’s age, education, occupation (working and employed), breastfeeding practices such as colostrum feeding, and exclusive breastfeeding practice. Maternal age was grouped as 15 to 20 years, 21 to 29 years, and 30 and above years. The mother’s education status was categorized as illiterate, literate, primary, secondary, and higher education. Early initiation of breastfeeding indicator included two categories i) within an hour (mother who breastfeed children within an hour of birth) and ii) delayed (mother who breastfeed children after 1 hour of birth).

### Data collection and analysis

Face to face interview was conducted with a mother of eligible children by the trained enumerators using a paper-based structured questionnaire. Legibility and completeness of data were ensured during the data collection period and any inconsistencies were addressed during the fieldwork. Anthropometric tools, SECA digital weighing scale for weight and height board (Stadiometer) for height measurement of 6 to 59 months children were used. Shakir tape was used to measure MUAC from the child’s left arm to the nearest 0.1 cm (1 mm) margin. Child’s weight was measured with no or minimum layer of dress and all the measurement was taken during daytime as recommended by WHO 2006 growth standards [32]. Descriptive statistics of 398 children aged 6 to 59 months were presented as frequencies and percentages along with the calculation of Pearson’s chi-square test to determine associations between predictors and outcome variable. Also, we used a multivariate logistic regression analysis to report the association between SAM and its determinants. All characteristics associated (*p* < 0.05) with outcome in chi-square test were included in the multivariable model. Odds ratios (OR) and 95% confidence intervals (CI) were derived and two-sided *p*-values less than 0.05 were considered as the statistical significance. No multicollinearity between independent variables was found. All analysis was performed in Stata software version 15.0 [33].

### Ethical considerations and informed consent

The ethical approval for this study was granted by the Nepal Health Research Council, Kathmandu, Nepal. Eligible mothers of under-five years children who were willing to participate in the study were interviewed after obtaining written consent.

## Results

Out of total 398 children of 6 to 59 months, more than half were female, and the average age of children in the study was 24.3 (±15.4) months. Almost 6% of children were severely malnourished and the mean MUAC of children in SAM category was 112.0 mm (±3.19). A higher percentage of children were from urban municipalities (59.3%), and lived in a joint family (55.3%). The average family size was 5.7 (±2.6) and the mean number of children in a household was 2.1 (±1.3); averages of family members and the number of children were higher in SAM category. Nearly three-quarters of children belonged to low-income households. One-fourth of the children were Madhesi and approximately one-fifth (18.8%) had no toilet facility in their household. More than half (56.5%) of the mothers reported to have kitchen garden at their household. Nearly two-thirds (64.1%) of the households were food secure while 11.8% of the households were severely food insecure (Table 1). Approximately half (46.5%) of the children participated in our study were eldest. More than half (58.3%) of the mothers were between the age group 21–29 years followed by 30 and above (29.4%) years. Almost one-fifth (18.8%) of the mothers were illiterate while nearly one-fourth (24.4%) of mothers had higher-level education. Six out of 10 children were breastfed within an hour of birth and more than half (67.8%) children were exclusively breastfed.

**Table 1:** Selected sociodemographic characteristics of mothers of 6 to 59 months children (*n* = 398)

Characteristics	Total		Child with SAM		***P*** value
	N	(%)	No	Yes	
	398	100	94.2	5.8	
**Household factors**					
**Place of residence**					0.780
Urban Municipality	236	59.3	94.5	5.5	
Rural Municipality	162	40.7	93.8	6.2	
**Family type**					0.758
Nuclear	178	44.7	93.8	6.2	
Joint	220	55.3	94.5	5.5	
**Family size**					0.012
Average family size **(**±SD)	5.7 (±2.6)		**5.7 (**±2.7)	**6.5 (**±2.1)	
1–5	222	55.8	96.8	3.2	
5 or more	222	44.2	90.9	9.1	
**Number of children**					0.003
Average number of children **(**±SD)	2.1 (±1.3)		1.2 (±0.4)	1.5 (±0.5)	
1–2	296	74.4	96.3	3.7	
3 or more	102	25.6	88.2	11.8	
**Household income**					0.050
< average (Rs 30,121)	294	73.9	92.9	7.1	
>average	104	26.1	98.1	1.9	
**Ethnicity**					0.002
Relatively advantaged (Brahmin/Chhetri)	143	35.9	98.6	1.4	
Relatively disadvantaged (Janajati/Muslims)	155	38.9	94.2	5.8	
Madhesi and other unidentified)	100	25.1	88.0	12.0	
**Toilet facility**					< 0.001
Yes	323	81.2	96.3	3.7	
No	75	18.8	85.3	14.7	
**Land size (*n*** **= 205)**					0.227
Less than 0.5 ha	101	50.3	96	4	
0.5 or more ha	100	49.8	92	8	
**Kitchen garden**					0.009
Yes	225	56.5	96.9	3.1	
No	173	43.5	90.8	9.2	
**Food Insecurity**					0.008
Food secure	255	64.1	96.9	3.1	
Mildly food insecure	41	10.3	92.7	7.3	
Moderate food insecure	55	13.8	90.9	9.1	
Severely food insecure	47	11.8	85.1	14.9	
**Child factors**					
**Sex of child**					0.452
Male	186	46.7	95.2	4.8	
Female	212	53.3	93.4	6.6	
**Child age (months)**					0.018
6–11	97	24.4	91.8	8.2	
12–23	137	34.4	91.2	8.8	
24–59	164	41.2	98.2	1.8	
**Mean age in month (**±SD)	24.3 (±15.4)		24.8 (±15.6)	15.6 (±8.2)	
**Mean MUAC (mm) (**±SD)	138.14 (±14.6)		139.7 (±13.4)	112.0 (±3.19)	
**Birth Order**					0.003
1	185	46.5	97.8	2.2	
2	123	30.9	93.5	6.5	
3 or more	90	22.6	87.8	12.2	
**Birth interval (*n*** **= 228)**					0.168
Less than 2 years	57	25.3	87.7	12.3	
More than 2 years	168	74.7	93.5	6.5	
**Maternal level factors**					
**Mother’s age at child birth (years)**					0.123
15–20	49	12.3	89.8	10.2	
21–29	232	58.3	93.5	6.5	
30 and above	117	29.4	97.4	2.6	
**Mother’s education**					0.022
No education	75	18.8	90.7	9.3	
Literate	46	11.6	87	13	
Primary	63	15.8	92.1	7.9	
Secondary	117	29.4	97.4	2.6	
Higher	97	24.4	97.9	2.1	
**Early initiation of breastfeeding**					0.722
Delayed	159	40.0	93.7	6.3	
Within an hour	239	60.1	94.6	5.4	
**Exclusive breastfeeding**					0.013
Yes	270	67.8	92.2	7.8	
Less than six months	128	32.2	98.4	1.6	

*p value are based on the Pearson chi-square test

In the multivariable logistic regression analysis, households with family size of five or more members was significantly associated with SAM [adjusted odds ratio (AOR): 3.96; 95% CI: 1.23–12.71]. Children of households experiencing severe food insecurity were four times (CI: 1.12–14.61) more likely to be severely malnourished compared to children in the food secure households. Three household indicators; children from Madhesi ethnicity, family without toilet facility, and no kitchen garden were significant predictors of SAM as reported in the unadjusted logistics regression model. Relative to 24 to 59 months children, children 6 to 12 months and 12 to 23 months had 12.1 and 6.6 times higher odds of being severely malnourished, respectively. However, there was no association with SAM and ethnicity, toilet facility, having kitchen garden, birth order, mother’s education level, and exclusive breastfeeding practices in the adjusted model (Table 2).

**Table 2:** Predictors of severe acute malnutrition among children 6 to 59 months visiting Outpatient Therapeutic Care Centers (*n* = 398)^a^

Characteristics	Unadjusted odds ratios	95% CI	***P*** value	Adjusted odds ratios	95% CI	***P*** value
**Family size**						
1–5 member/s (Ref)	1			1		
5 or more members	3.07	(1.24–7.64)	0.02	3.96	(1.23–12.71)	0.02
**Number of children**						
1–2 (Ref)	1			1		
3 or more	3.46	(1.47–8.10)	0.004	4.92	(0.24–100.1)	0.30
**Ethnicity**						
Relatively advantaged (Brahmin/Chhetri) (Ref)	1			1		
Relatively disadvantaged (Janajati/Muslims)	4.35	(0.92–20.47)	0.06	1.20	(0.12–12.23)	0.88
Madhesi and other unidentified	9.61	(2.10–43.98)	< 0.01	3.18	(0.30–33.84)	0.34
**Toilet facility**						
Yes (Ref)	1			1		
No	4.45	(1.88–10.54)	< 0.00	1.54	(0.48–4.96)	0.47
**Kitchen garden**						
Yes (Ref)	1			1		
No	3.17	(1.28–7.90)	< 0.01	2.56	(0.82–8.00)	0.11
**Food Insecurity**						
Food secure (Ref)	1			1		
Mildly food insecure	2.44	(0.62–9.59)	0.20	1.30	(0.26–6.53)	0.75
Moderate food insecure	3.09	(0.97–9.83)	0.06	0.58	(0.13–2.52)	0.46
Severely food insecure	5.40	(1.86–15.72)	< 0.01	4.04	(1.12–14.61)	0.03
**Child factors**						
**Child age (months)**						
6–12	4.82	(1.25–18.64)	0.02	12.10	(2.06–71.09)	< 0.01
12–23	5.15	(1.42–18.65)	< 0.01	6.61	(1.24–35.25)	0.03
24–59 (Ref)	1			1		
**Birth Order**						
1 (Ref)	1			1		
2	3.15	(0.93–10.69)	0.066	4.09	(0.99–16.87)	0.05
3 or more	6.30	(1.95–20.39)	< 0.01	0.64	(0.03–15.14)	0.78
**Maternal factors**						
**Mother’s education**						
No education	4.89	(0.99–24.27)	0.05	0.34	(0.026–4.59)	0.42
Literate	7.13	(1.38–36.82)	0.02	0.57	(0.04–7.80)	0.68
Primary	4.10	(0.77–21.80)	0.10	0.91	(0.08–10.18)	0.94
Secondary	1.25	(0.21–7.64)	0.81	0.54	(0.052–5.61)	0.61
Higher (Ref)	1			1		
**Exclusive breastfeeding**						
Yes (Ref)	1			1		
No	0.19	(0.043–0.82)	0.03	0.38	(0.070–2.03)	0.26

*Ref.* Reference Category, Statistically significant at *p* < 0.05

^a^ Estimates are adjusted odds ratios and 95% confidence intervals from logistic regression

## Discussion

Our study used WHO standards for MUAC cut-off of below 115 mm to identify SAM among children aged 6 to 59 months. The MUAC measurement was used over weight-for height below − 3 SD of the WHO standards because both of these measures give almost similar prevalence of SAM [3]. MUAC is regarded as a cost-effective and easiest method to early detect SAM in children with minimal risk and many potential benefits [34]. We found that only 5.8% of children admitted to the OCCTs were severely malnourished. Significant predictors of SAM among children aged 6 to 59 months included family size, household access to food, and child age. The study further indicated that the prevalence of SAM was significant (*p* < 0.05 in Chi-square test and in the unadjusted regression model) among families having three or more children, children belonging to Madhesi ethnicity, households with no toilet facilities and no kitchen garden, order of birth greater than two, illiterate mothers and children with no exclusive breastfeeding practices.

Households with five or more members and households with three or more children were statistically associated with SAM. Higher the number of family members in the household more will be the burden to households to provide optimum nutritious food to all the family members and children. Higher the number of children in households, it unlikely that every child gets proper care and time, putting them at a higher risk of being malnourished. This finding is coherent with a study conducted in India [35], Ethiopia [36, 37], and Uganda [38]. Our study finding indicated that SAM among children was significantly associated with ethnicity. Children belonging to the Madhesi were higher at risk of being malnourished indicating disparities in nutrition status among children in Nepal. A study investigated the inequality in terms of ethnicity in the utilization of health services in Nepal reported a higher prevalence of childhood malnutrition among Terai ethnicities where mainly Madhesi ethnicity resides [17]. This may be due to the fact that Madhesi ethnicity is an underprivileged group and have socio-cultural practices that hinder health service utilization and the adoption of healthy practices. In our study toilet facility at household showed a significant association with SAM. The availability of toilet facility is directly linked with child’s hygiene and nutrition. Sanitation and hygiene behaviours, therefore, are essential factors to improve the nutritional status of children. Similar results were reported in studies done previously [39, 40]. Children from the food insecure households were more likely to be malnourished. Several studies have found a similar association between household food insecurity and malnutrition among children [41, 42]. This finding is plausible as the reduced or compromised quality of diet due to lack of food or limited availability of food, could not meet the dietary requirement in terms of quantity and quality of child’s diet. This poses children at a greater risk of undernutrition [43]. Notably, among mothers who were illiterate, the likelihood of children being severely malnourished was higher. The unadjusted model in our study reported significant association between SAM and mother education. This finding is consistent with the studies from Bangladesh [9] Ethiopia [44], and Pakistan [11] which showed that maternal education is the key factor by which mothers have a better understanding of child nutrition and acts as a protective factor against undernutrition. Exclusive breastfeeding practice in our study was significantly associated with SAM. This finding was comparable with the studies from Nepal and Pakistan [11, 45, 46]. The finding highlights the importance of exclusive breastfeeding from which children receive the nutrition that has several benefits including a reduction in gastrointestinal infection [47]. However, the association was not found between SAM and early initiation of breastfeeding. The possible explanation for this would be the exclusive breastfeeding rather than early breastfeeding provides protective nutrition to the children for an extended period of time. This finding is in agreement with findings from Nepal and India [45, 46, 48].

Apart from these findings, this study has several limitations. The cross-sectional design of the study limits to capture the actual prevalence of acute malnutrition among children and the observational study design restrained us to eliminate associations are due to residual confounding. Since the study was conducted in two districts of province 1 and 2 in Nepal, the findings are not a true representation of acute malnutrition of children of the entire country.

## Conclusions

In this study, notably higher percentages of SAM children were from households with five or more family members. We found that household food access and child’s age were independently associated with acute childhood malnutrition. Hence, education and awareness to the mothers could play a vital role in improving child nutrition status. Our findings emphasized the need to strengthen the nutrition status of children in food-insecure households by promoting access to food and various source of nutrition. Nutrition programs should be prepared to serve the poorest and disadvantaged mothers and children during and after an emergency. Large-scale epidemiological studies are needed to investigate the determinants of SAM and explore the best approach to deal with SAM during emergencies.

## Data Availability

The datasets analysed in this study are not publicly available due to ethical concerns.

## Abbreviations

AOR: Adjusted Odds Ratio
CI: Confidence Interval
IMAM: Nepal Integrated Management of Acute Malnutrition
NDHS: Nepal Demographic and Health Survey
MUAC: Mid-upper arm circumference
OTCC: Outpatient Theraputic Care Centers
MAM: Moderate Acute Malnutrition
RUFT: Ready-to-use therapeutic food
SAM: Severe Acute Malnutrition
SD: Standard Deviation
WHO: World Health Organization
